# Origin of the Acoustic Bandgaps in Hypersonic Colloidal Phononics: The Role of the Elastic Impedance

**DOI:** 10.1021/acs.jpcb.2c03923

**Published:** 2022-08-23

**Authors:** Yu Cang, Rebecca Sainidou, Pascal Rembert, Giulia Magnabosco, Tim Still, Nicolas Vogel, Bartlomiej Graczykowski, George Fytas

**Affiliations:** † Max Planck Institute for Polymer Research, Ackermannweg 10, 55128 Mainz, Germany; ‡ School of Aerospace Engineering and Applied Mechanics, Tongji University, Zhangwu Road 100, Shanghai 200092, China; § Laboratoire Ondes et Milieux Complexes UMR CNRS 6294, UNIHAVRE, Normandie University, 75 rue Bellot, F-76600 Le Havre, France; ∥ Institute of Particle Technology, Friedrich-Alexander University Erlangen-Nürnberg, 91058 Erlangen, Germany; ⊥ Faculty of Physics, Adam Mickiewicz University, Uniwersytetu Poznanskiego 2, Poznan 61-614, Poland; # Institute of Electronic Structure and Laser, FO.R.T.H, N. Plastira 100, /0013, Heraklion 71110, Greece

## Abstract

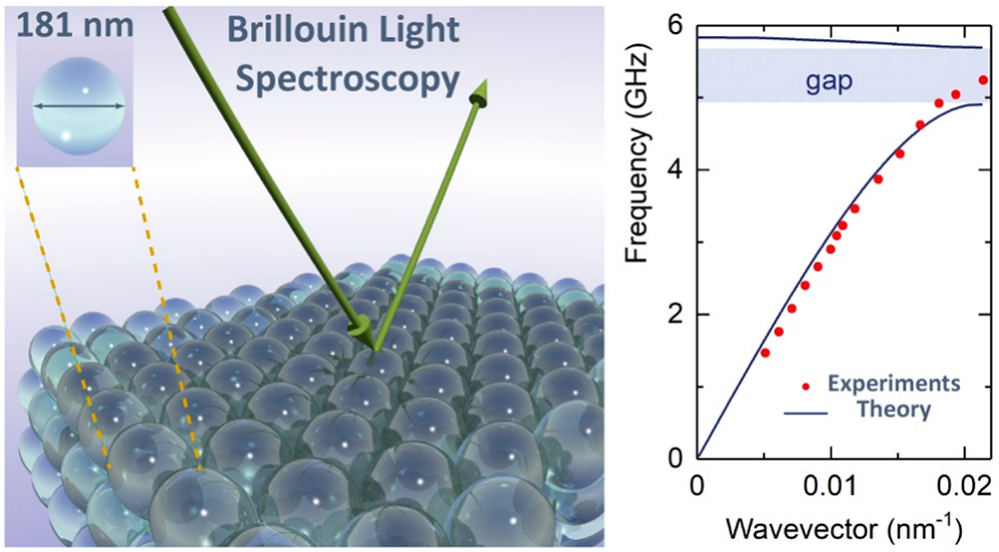

How phonons propagate in nanostructures determines the flow of elastic and thermal energy in dielectric materials. However, a reliable theoretical prediction of the phonon dispersion relation requires experimental verification both near to and far from the Brillouin zone of the nanostructure. We report on the experimental hypersonic phonon dispersion of hard (SiO_2_) and soft (polymer) fcc colloidal crystals infiltrated in liquid polydimethylsiloxane with different elastic impedance contrast using Brillouin light spectroscopy. We discuss the distinct differences with first-principles full elastodynamic calculations involving a multiple-scattering theory. Interparticle contacts strongly impact the long-wavelength speed of sound and the nature of the particle vibration resonance-induced hybridization hypersonic bandgap. The absence of the order-induced Bragg bandgap in SiO_2_ and its presence in soft opals cannot be fully accounted for by the theory, limiting its predictive power. Bridging the elasticity of the two colloidal crystals with suitable SiO_2_ core–shell (polymer) particles reveals an unprecedented crossover behavior in the dispersion relation. In view of many conversational parameters, the control tuning of phonon propagation in soft matter-based hypersonic phononics remains challenging.

## Introduction

The propagation of acoustic (elastic) waves in architected matter is a generic problem that impacts material and life sciences as the phonon senses both the bulk and the surface of the matter.^1,2^ Phonon propagation in composite structures depends on many conversational parameters (three per solid component).^3^ This number increases further when anisotropy is introduced in the structure design.^4^ Besides the structural and elastic parameters, the phononic material behavior, i.e., the controlled flow of elastic waves, is further influenced by additional factors such as complex structural dynamics,^5^ spatial confinement, and interfacial effects.^6,7^ There is, therefore, rich, unexplored, and hardly predictable fundamental science that needs a supporting foundation to be established.^8,9^ The key quantity is the phonon dispersion relation ω(*k*), which relates the angular frequency ω and the wavenumber *k* of the propagating elastic wave in the composite material. Engineering of ω(*k*) to allow elastic wave propagation only for desired frequencies, polarizations, and directions requires control of structure periodicity, component dimensions, and elastic parameters. The extension to high-frequency phononics to enable simultaneous manipulation of hypersonic phonons (GHz range) and visible light (400–800THz), and signal processing in wireless communication devices, needs organization in the submicron and nanometer scale range via self-assembly and nanofabrication techniques.^2^ The required organization at the submicron scale is a ubiquitous property of soft matter that allows such fabrication of structures with manifold functionalities. Control over the phonon dispersion can impact the flow of elastic waves, strength, and toughness concomitantly, and heat transport in dielectric hybrid materials.^9^

Experimental access to ω(*k*) at GHz frequencies can be provided only by Brillouin light spectroscopy (BLS), however, for sufficiently transparent structures.^10,11^ For nontransparent samples, thermally stimulated phonon techniques such as time-resolved picosecond ultrasonics,^12^ laser-induced transient grating,^13^ and a recently reported frequency-domain hybrid technique^14^ have been employed. However, the *k*-accessibility is either limited to 1D phononics or restricted to surface or plate/membrane acoustic waves. For 1D hypersonic phononics, the theoretical ω(*k*) is well-documented, revealing an interference Bragg (BG) bandgap along the periodicity direction,^15^ while for the 2D structures for the two symmetry directions, the theoretical ω(*k*) is much richer than that experimentally recorded.^8^ For the dispersion of surface Lamb waves in 2D colloidal monolayers, however, the theory represents the experiment well.^16,17^ The phonon propagation in 3D structures is the least understood in view of the number of involved parameters,^10,18,19^ such as the viscoelastic nature of the matrix and the different symmetry directions, but also due to the limited experimental evidence.^20^

3D structures formed by submicron colloidal particles have unique advantages due to facile self-assembly fabrication, variation of the volume fraction,^21^ and the host of localized mechanical resonances.^22^ There is a handful of experiments based either on soft (polymeric) or hard (inorganic) nanoparticles (NPs) using BLS^22−24^ and pump–probe^25,26^ techniques. While only the former technique records ω(*k*) with direct observation of the BG along the high-symmetry direction,^18^ the latter provides indirect evidence of a bandgap.^27^ In addition to the order-induced BG, a second bandgap robust to disorder opens up in the ω(*k*) of polystyrene (PS) and poly(methyl methacrylate) (PMMA) colloidal opals infiltrated with a liquid to warrant optical transparency.^19^ The latter feature, termed hybridization gap (HG), is assigned to the quadrupolar (*l* = 2) NP resonance in the case of a liquid matrix.^19^ However, the theory cannot represent the experimental ω(*k*) both near to and far from the Brillouin zone as it underestimates the effective-medium sound velocity at low wavenumbers. Boosting the elastic impedance contrast exemplified for SiO_2_ opals has a strong impact on ω(*k*) unanticipated from the corresponding low-contrast opals, already expected by theoretical predictions in 3D fcc arrays of soft and hard colloids, made of almost touching, close-packed NPs.^28^ In this work, we utilized BLS to record the ω(*k*) of hard SiO_2_ opals with different diameters and SiO_2_–PMMA core–shell opals with the same SiO_2_ core diameter and different PMMA shell thicknesses in order to bridge with the soft PMMA opals. The experimental band diagrams of the soft and hard colloid opals both infiltrated in liquid polydimethylsiloxane (PDMS) are distinctly different with two and single bandgaps, respectively. The low-frequency HG is assigned to the hybridization of the sphere’s dipole *l* = 1 mode and not the earlier proposed *l* = 2, assuming a liquid matrix in the close-packed colloids.^19^ The effective-medium longitudinal sound velocity exceeds the theoretical value for solid/liquid matrix phononics, suggesting a granular-type consolidation. The paper is organized as follows: after a short description of the methods, the experimental and theoretical band diagrams of the SiO_2_ and SiO_2_–PMMA core–shell colloidal crystals infiltrated with PDMS are presented and discussed in the two subsections of the Results and Discussion section.

## Methods

### Fabrication of Wet Opals

The colloidal crystalline films were fabricated by vertically lifting the glass substrates from the aqueous colloid dispersion with subsequent fluid infiltration. Bare SiO_2_ and core–shell SiO_2_–PMMA particles of diameter *d* were used for the fabrication of the opals, whereas PDMS was selected as a medium to infiltrate the particle opals. Besides crystalline films, the noncrystalline hybrid films comprised of binary SiO_2_–PMMA particles with two different diameters were prepared in the same fashion. For the particle preparation, the SiO_2_ particles with diameters from 143 to 375 nm were synthesized via the Stöber method. The SiO_2_ particles were then coated with the softer PMMA shells by emulsion polymerization.^29^ The PMMA shells were tailored with three different thicknesses (25, 57, and 112 nm) on the SiO_2_ cores with a diameter of 181 nm, leading to the core–shell (SiO_2_–PMMA) particles with diameters *d* ranging from 232 to 405 nm. Two silica opals with diameters 219 and 375 nm are prepared by ultracentrifuging the silica/liquid ethoxy–ethoxyethyl acrylate (SR256) dispersion (34 vol % silica). The obtained polycrystalline samples are close-packed silica particles in an SR256 matrix with a volume fraction of about 74%.

### Brillouin Light Spectroscopy (BLS)

BLS is a powerful optical technique to probe the phonon propagation at GHz frequencies and hence access the hypersonic phonon band structure. Utilizing the photoelastic interactions between incident light and thermally activated phonons, BLS records the spectra of inelastically scattered light by phonons with wave vector **q**, which equals scattering wave vector **k**. The **q** = **k_i_** – **k_s_** along a specific direction could be selected by scattering geometry, with **k_i_** and **k_s_** being the wave vector of the incident and scattered light, respectively. The spectra consist of a single doublet with the Doppler shifts of 2*πf = ±cq* at the low-**q** regime, where *c* is the phase velocity of longitudinal (or transverse) phonons selected by the input polarizer V(V) and output analyzer V(H) with V and H being the vertically and horizontally polarized light, respectively. The transmission geometry is employed in this work that allows the  to vary up to 0.024 nm^–1^, where the incident angle α is half of the scattering angle and λ = 532 nm is the wavelength of incident light. The high *q*-range, up to about 0.035 nm^–1^, is accessible in the reflection geometry, where knowledge of the medium refractive index is required.^11^

### Theoretical Calculations

The dispersion relation ω(*q*) of the elastic eigenmodes (band structure) in these colloidal assemblies of spherical particles is obtained by applying a first-principles full elastodynamic multiple-scattering theory.^30,31^ It takes into account all interactions of the multipole expansions of the elastic field between particles, assuming they are nonoverlapping, almost touching, for close-packed structures. Density-of-states (DOS) calculations^32^ are also performed for individual particles embedded in a host matrix or for monolayers of such interacting particles. Thus, the additional virtual bound states induced in these systems with respect to those of the infinitely extended host matrix are deduced. All band structure calculations concerning the fcc colloids in this study were performed along the [111] (ΓL) direction, which is very close to the ΓM direction that better describes the experimental setup.^18^ This choice does not affect the general picture obtained (without loss of generality, small deviations are expected between the dispersion diagrams along these directions^10^), but it strongly facilitates the numerical computation, since it becomes cumbersome along ΓM for very compacted (close-packed) structures. For systems organized in a fcc structure with a solid host matrix surrounding the spherical particles, three kinds of bands exist along the [111] direction: transverse bands which are doubly degenerate (Λ_3_ symmetry), longitudinal bands which are nondegenerated (Λ_1_ symmetry), and deaf (inactive) bands which are nondegenerated (Λ_2_ symmetry).^10^ In the case of a fluid host matrix and for the same crystalline structure and direction of BZ, transverse modes cannot exist, and hence, doubly degenerate bands (Λ_3_ symmetry) become deaf. Classification of bands is realized using group theory arguments in combination with (a) the calculation of the transmittance of an elastic wave through a finite slab of the crystal and (b) an analysis of the eigenmodes in the plane wave representation basis (for more details, the reader can refer to previous works^10,28,30,31^).

## Results and Discussion

### Silica Colloidal Crystals

**Figure 1:**
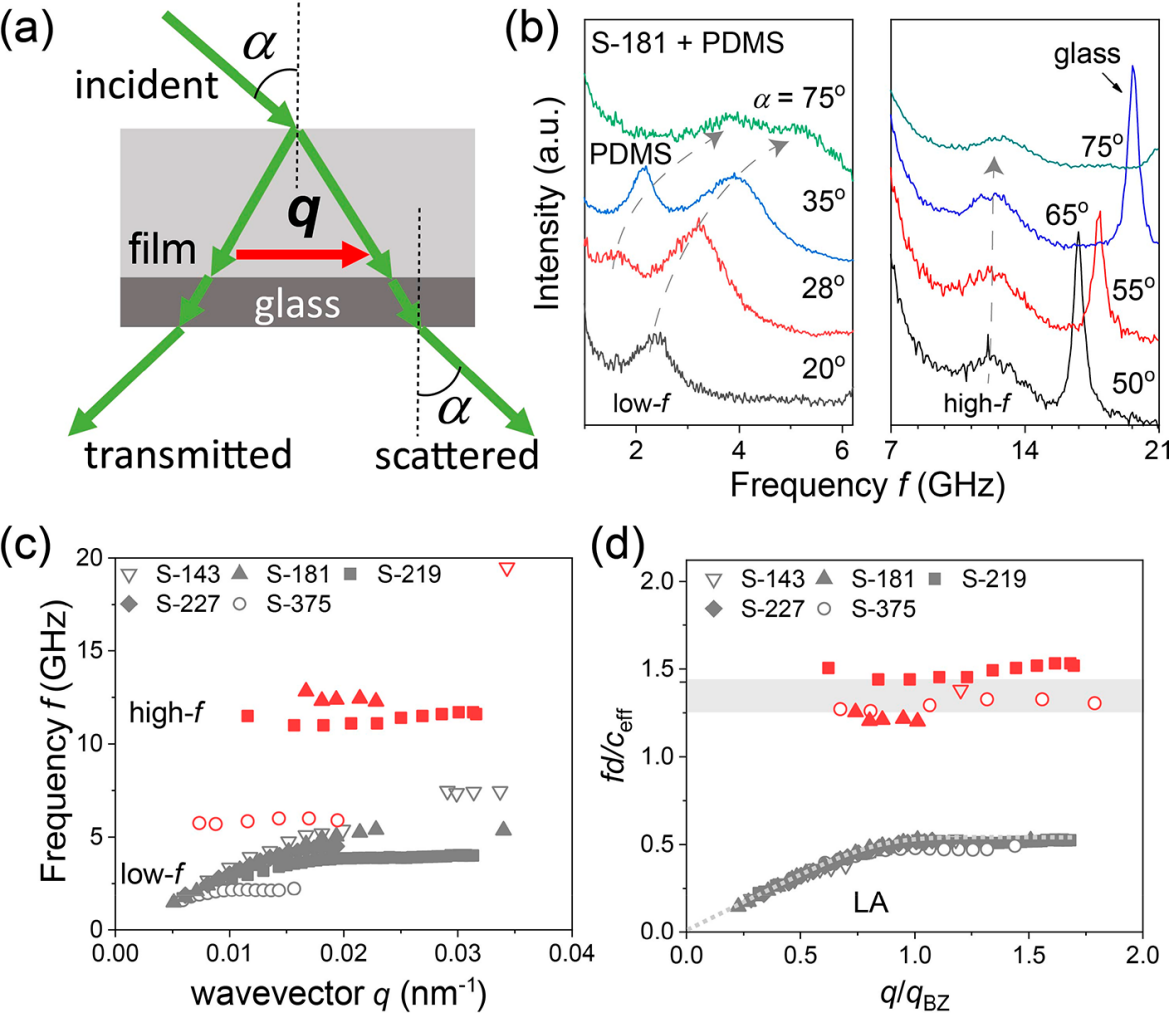
Phonon band diagram of SiO_2_ fcc opals infiltrated with PDMS. (a) Scheme of the transmission scattering geometry: the wavevector **q** is directed parallel to the film plane when the incident angle α is half of the scattering angle. (b) Representative anti-Stokes BLS spectra of the glass-supported SiO_2_ fcc colloidal crystals (S-181, with particle diameter, *d* = 181 nm) infiltrated with PDMS at a different α recorded at low (left) and high (right panel) free-spectral-range. (c) The recorded dispersion relations *f*(*q*) for SiO_2_ crystalline films with SiO_2_ diameter between 143 and 375 nm. (d) The corresponding normalized dispersion relations, *fd*/*c*_eff_ vs *q*/*q*_BZ_, where *c*_eff_, the effective-medium sound velocity, is obtained from the linear fit at low *q* (panel c) and  is the edge of the first Brillouin zone (BZ) in the ΓM direction. The dashed lines and shaded area are guides to the eye.

First, we examine the phonon propagation in SiO_2_ colloidal crystals infiltrated with low-viscosity PDMS (*M*_n_ = 980) fluid on the account of its high elastic contrast and similar refractive index with silica enabling the crystals to be optically transparent in the visible range. The dispersion relation, *f*(*q*), was recorded using the transmission BLS geometry with the phonon wave vector **q** parallel to the film (Figure 1a). Polarized (VV) BLS spectra recorded at different α values (and hence *q*’s) are shown in Figure 1b for two spectral ranges to resolve low-frequency (low-*f*) and high-frequency (high-*f*) phonons. For *qd* < 1, the systems appear homogeneous, and only the effective-medium longitudinal acoustic (LA) phonon (low-*f* mode indicated by the second arrow in the left panel of Figure 1b) is resolved; the first arrow indicates the LA phonon in the PDMS layer atop the colloidal crystal. The BLS spectrum is represented by a sum of Lorentzian line shapes with adjustable parameters, the amplitude, frequency at the peak position, and line width for each spectral line. A peak is characterized as single even if it is broad as judged from the random deviation plot. At low *q*’s, the frequency of the LA phonon, *f*_LA_, in the SiO_2_ opals is expected to be proportional to *q*, but it reaches a plateau value *f*_bend_ at higher *q*’s (Figure 1c for different *d*). Both the asymptotic value and the range of the linear dependence, *f*_LA_ = *c*_eff_*q/*2π, increase with a decreasing SiO_2_ diameter. For five opals with different SiO_2_ diameters, a successful superposition of this LA branch is obtained in the plot *f*_LA_*d*/*c*_eff_ vs *q*/*q*_BZ_ in Figure 1d, where  along the ΓM direction. The deliberately chosen direction cannot be experimentally inferred, but bandgaps are anticipated along high-symmetry directions.^18,19^ In this representation, the asymptotic *f*_LA_*d*/*c*_eff_ occurs at *q*/*q*_BZ_ ≈ 0.5. Note that S-219 and S-375 infiltrated opals^33^ display a lower *c*_eff_ value than S-143 and S-181 (4th column in Table 1) due probably to a weaker interface connectivity, as both the fluid matrix and fabrication procedure differ (Methods section).

**Table 1:** Characteristics of SiO_2_ and Core–Shell (SiO_2_–PMMA) Infiltrated Opals, Deduced from Experiments

sample code (S-*d*, S-P-*d*)^a^	particle diameter *d* (nm)	SiO_2_ volume fraction ϕ^c^	effective-medium sound velocity in the PDMS-infiltrated opals *c*_eff_ (m/s)^d^	particle vibration in air *f*(1, 2) (GHz)	particle vibration in PDMS *f*(1, 2) (GHz)
S-143	143	1	2020	19.5	19
S-219	219	1	1670^e^		11.2^e^
S-227	227	1	1960		
S-375	375	1	1690^e^		5.9^e^
S-181	181	1	1980	13^g^	12.4
S–P-232^b^	232	0.47	1800	10.3^g^	9.4
S–P-294^b^	294	0.23	≤1530^f^	6.3^g^	5.3
S–P-405^b^	405	0.09	≤1560^f^	3.9^g^	3.8

a S: SiO_2_. P: PMMA. *d*: particle diameter.

b *d*_SiO2_ = 181 nm.

c ϕ = (*d*_SiO2_/*d*)^3^.

d Error ±2%.

e Particles in liquid SR256 matrix^33^

f *q*-range not sufficiently low

g Ref (34).

The BLS spectra for the S-181 colloidal crystal (right panel of Figure 1b) show a second high-*f* phonon with a virtually *q*-independent frequency, *f*_LO_; the strong sharp peaks are due to the LA phonon in the glass substrate. According to Figure 1c, the value of *f*_LO_ increases from 5.9 to 19.5 GHz with a decreasing SiO_2_ diameter from 375 to 143 nm. Triggered by this dependence, *f*_LO_ is about 10% red-shifted compared to the quadrupolar resonance frequency *f*(1, 2) = A*c*_T_,_SiO2_/*d*_SiO2_ of the corresponding SiO_2_ nanoparticle in air (see Table 1 and Figure S1), where *A* is constant (≈0.85) and *c*_T_,_SiO2_ is the transverse sound velocity in SiO_2_ nanoparticles. Consequently, a superposition of the *f*_LO_ values is anticipated in the plot of Figure 1d if *c*_T,SiO2_ is assumed to have a similar value in all SiO_2_ nanoparticles, i.e., similar porosity. In fact, *f*_LO_*d*/*c*_eff_ ≈ 1.3 within ±5% resulting in a huge bandgap of a width Δ*f*/*f*_mid_ ≈ 0.5 relative to the middle frequency of the gap, *f*_mid_. Note that *f*(1, 2) of the SiO_2_ particles in air becomes only slightly softer than in PDMS due to the large elastic impedance (see Figure S1). In addition to *f*(1, 2), the bending of the LA at *f*_bend_ is also superimposed in the reduced plot of *fd*/*c*_eff_ in Figure 1d and will be rationalized in the next section.

**Figure 2:**
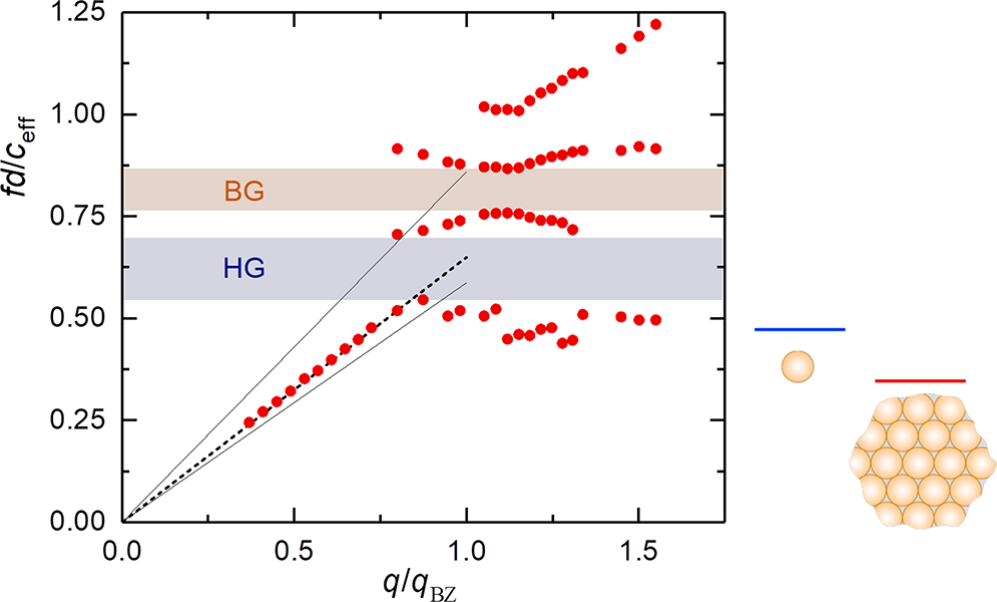
Band diagram of soft colloid-based hypersonic phononics. Experimental phonon dispersion of a close-packed colloidal crystal consisting of PMMA spherical particles with diameter *d* = 327 nm infiltrated with fluid PDMS as recorded by BLS at ambient conditions. Both *x*- and *y*-axes are reduced dimensionless wavevector and frequency, respectively. The dashed line in the low *q* linear regime denotes the experimental *c*_eff_ = 1720 m/s. The two thin lines corresponding to *c*_eff_ = 1550 m/s and c_eff_ = 2270 m/s refer to the theoretical predictions for fcc arrays of close-packed PMMA spheres along ΓL direction in liquid PDMS (*c*_L_ = 1050 m/s, Figure 3a) and spherical liquid-PDMS pockets (*d*_c_ = 231 nm) in PMMA (inverse topology, Figure 3c). The hatched stripes indicate the periodicity-induced Bragg gap (BG) and the hybridization bandgap (HG) associated with PMMA resonances. The two short horizontal lines in the right margin denote the frequency of the quadrupolar (*l* = 2) mode for a single PMMA sphere (*d* = 327 nm) in liquid PDMS (blue line) and the resonant frequency resulting from the interactions of an fcc (111) plane of spheres (red line), as obtained from DOS calculations.

In contrast to the hard-sphere (SiO_2_) colloid-based phononics, their soft (and low-density) PS and PMMA counterparts have revealed the presence of two bandgaps.^19^ The experimental phonon band diagram of a PMMA-based hypersonic phononic crystal also infiltrated with PDMS is qualitatively different from the SiO_2_ case, as shown in Figure 2. The dispersion is rich with two phononic bandgaps (BG and HG) clearly resolved (hatched stripes in Figure 2). We should note that the observation of bandgaps in the BLS experiment is unique because of the clear splitting of the spectral lines, irrespective of the line broadening (BLS spectra in Figures 1b and 4b, and refs (18 and 19)). The high-frequency bandgap was interference-induced BG associated with the structure periodicity. The bandgap at a lower frequency was assigned to the particle spheroidal Lamb mode (1,2) and is robust to the structure disorder.^19^ While the assignment of the higher-frequency BG seems unique, as a periodicity is prerequisite, the association of the HG at a lower frequency to the particle quadrupolar (*l* = 2) mode in a liquid matrix is questionable. The particle vibration frequency, *f*(1, 2), in liquid PDMS is about 30% lower than the frequency *f*_HG_ in the middle of the HG as indicated by the blue line in the margin of Figure 2. However, for an ensemble of such (unconnected but close-packed) spheres arranged in an fcc (111) plane, these individual modes are combined to give rise to a red-shifted collective resonant mode, as predicted by DOS calculations (red line in the margin of Figure 2). This mode, which should be the fingerprint of the corresponding HG, is predicted at a much lower frequency than the experimental *f*_HG_.^19^ Hence, the assumption of a liquid-PDMS matrix and/or unconnected soft colloidal particles does not confirm the experimental trend for the *f*_HG_ in the PMMA opal, also observed in PS opals.^16^

**Figure 3:**
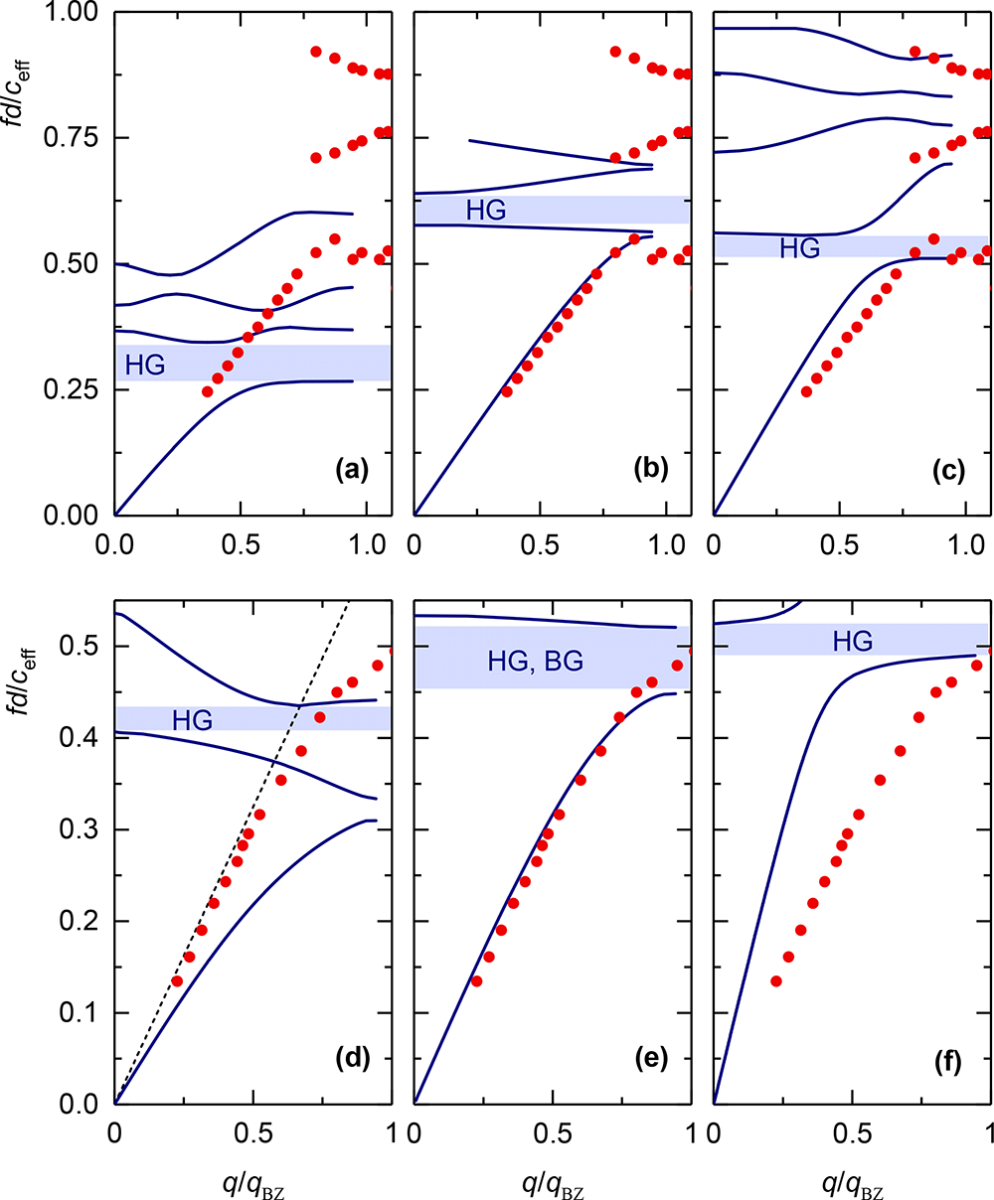
Band diagram of soft and hard colloid-based hypersonic phononics. Theoretical dispersion for longitudinal bands (solid lines) for fcc arrays of close-packed PMMA (*d* = 327 nm) in panels a–c and SiO_2_ (*d* = 181 nm) in d-f particles infiltrated with PDMS considered either liquid (*c*_L_ = 1050 m/s) in panels a and d or solid (*c*_L_ = 1050 m/s, *c*_T_ = 400 m/s) in panels b and e. For liquid PDMS, the long-wavelength linear parts of the calculated dispersion plots shown in panels a and d correspond to slopes *c*_eff_ = 1550 and 1480 m/s, respectively, for PMMA and SiO_2_ opals. For solid PDMS, the corresponding values are *c*_eff_ = 1910 m/s (b) and 2050 m/s (e). Panels c and f show the theoretical band diagram for spherical liquid-PDMS pockets (diameter *d*_c_ = 231 nm in PMMA and 128 nm in SiO_2_ inverse topology) for which *c*_eff_ = 2270 m/s (c) and 3790 m/s (f). From the experimental longitudinal acoustic branch (points), *c*_eff_ = 1720 m/s (PMMA opal in PDMS) and *c*_eff_ = 1980 m/s (SiO_2_ opal in PDMS).

The failure of the above model to capture the experimental data is further confirmed by examining the effective-medium *c*_eff_, obtained from the long-wavelength linear dispersion (dashed line in Figure 2). For the anticipated liquid nature of PDMS that does not support shear modes, the computed band diagram shown in Figure 3a fails to capture the main experimental quantities. In addition to the large deviation between the HG of the pure quadrupolar (*l* = 2) origin, predicted at much lower frequencies (red line in the margin of Figure 2) and the experimental *f*_HG_, the computed *c*_eff_ in the fcc PMMA opal in liquid PDMS is about 10% lower than the experimental value. The former is indicated, for comparison, as a thin line below the experimental linear acoustic dispersion (dashed line) in Figure 2. An increase of *c*_eff_ is feasible due to interaction-induced contacts between neighboring PMMA spheres, in analogy to granular systems.^35−37^

The experimental *c*_eff_ can be represented either by increasing *c*_L,PDMS_ or introducing a nonvanishing *c*_T,PDMS,_ which means solidification of PDMS.^38^ However, none of these possibilities can be justified in the absence of spatial confinement in the nanometer length scale. More realistic is a granular structure that can increase *c*_eff_ due to contact formation between close-packing particles.^36,39,40^ Evidence of a transformation from a point–point contact to a line–line contact between PS particles when the dry opal is infiltrated with PDMS is discernible in the SEM images.^18^ The presence of interactions is clearly manifested in the particle elastic vibrations with a split of (1, 2) mode and the presence of a new lower-frequency rattling mode (1, 1).^24,41−43^ In this context, the experimental *c*_eff_ can be represented by assigning an effective *c*_T,PDMS_ = 400 m/s that was used to compute the band diagram in Figure 3b. The calculated band diagram is strongly affected by the nature (fluid or solid) of the host matrix: a new HG opens up very close to the experimental reduced frequency, though narrower than the one observed experimentally in Figure 3b. It originates from dipole (*l* = 1) modes of the individual particles, which interact to form a localized narrow band, leading to anticrossing with the effective-medium longitudinal phonon.^16,44^ However, there is no indication of a BG along the ΓL direction in the theoretical band diagram. We have further examined the assumption of a consolidated crystal in the case of contacts between the PMMA spherical particles, modeled by an inverse-topology scheme, i.e., liquid-PDMS inclusions (considered spherical for simplicity) in a solid PMMA matrix. The computed band diagram in Figure 3c still predicts an otherwise narrow HG originating from dipole (*l* = 1) modes of the individual cavities centered at about 0.51 in reduced frequency units. More importantly, a second narrow gap of BG type opens up at about 0.81, coinciding with avoided-crossing effects at the same frequency region. These theoretically predicted gaps semiquantitatively capture the two mainly observed experimental gap regions, of HG and BG origin, centered, respectively, at ∼0.62 and ∼0.82 in the normalized diagram of Figure 2, though this scenario overestimates *c*_eff_ by 23%.

The theoretical band diagram is extremely sensitive to the topology (crystalline structure and degree of interface connectivity) of the soft colloidal crystals and the state of the infiltrated matrix (Figure 3a–c). However, none can represent the complete experimental dispersion recorded for inhomogeneously interacting colloidal particles allowing for both liquid pockets and a continuous liquid matrix with a possible impact on their spherical symmetry. It seems that the clearly resolved BG for periodic soft colloidal crystals is elusive in the theoretical band diagram of the considered structures. On the other hand, the origin of the HG is the hybridization of the sphere’s *l* = 1 mode and not the earlier proposed *l* = 2, invoking a predominantly solid matrix in the close-packed colloids.^19^ We note that in a less compact colloidal dispersion^45^ excellent agreement was found between the theoretical calculations assuming nonoverlapping close-packed particles and the experiment, confirming an HG relying on resonant modes of quadrupolar (*l* = 2) origin in the individual particle. The nature (fluid or solid) of the host matrix in which a given solid spherical particle is embedded plays a crucial role in the appearance of appropriate resonant states localized in the particle. When shear modes are supported in the outer region (solid host), the lowest-frequency resonant mode is dipolar (*l* = 1), while when shear modes are not supported in the host region (fluid matrix) the lowest-frequency resonant mode becomes quadrupolar (*l* = 2). This difference is certainly related to the different boundary conditions applied across the particle’s interface; we note in passing that piecewise boundary conditions, combining both solid and liquid phases of the host matrix on distinct domains of the same particle’s surface, cannot be considered by our theoretical method, though they could constitute an interesting approach. In similar systems consisting of fcc arrays of almost touching polystyrene spherical particles embedded in cross-linked PDMS (nonvanishing *c*_T_), BLS experiments have revealed the occurrence of a very narrow HG centered at about 4 GHz^46^ whose nature (relying on dipole resonances of individual particles) and frequency (position and width) characteristics are nicely predicted by our theoretical calculations (see Figure S2), assuming again an inverse-topology scheme (i.e., PDMS pockets in a solid PS matrix).

In the case of SiO_2_, interparticle contacts are still present, but the high elastic modulus should prevent particle deformation and the extent of contacts can be judged from the value of *c*_eff_. Moreover, *f*(1, 2) shifts to a much higher frequency than for the soft colloidal spheres of the same diameter due to the higher *c*_T,SiO2_ value. Figure 3d–f shows the theoretical band structure of a SiO_2_ opal with *d* = 181 nm infiltrated with PDMS for three distinct cases, liquid (panel d) and solid (panel e) PDMS host matrix and inverse topology in panel f, as for the PMMA opal case. Obviously, the liquid matrix assumption fails to describe the experimental dispersion plot in Figure 3d. The theoretical calculations assuming almost touching but nonoverlapping spherical scatterers show a typical dispersion structure already encountered in silica fcc colloids immersed in water-like fluid environments or in air.^28,47^ In this case, good agreement between theory and experiment was obtained in both, the effective-medium slope and the position and width of an HG (at about 0.4 in reduced frequency units in the plot of Figure 3d). The computed HG originates from collectively formed modes localized in the interstitial fluid-filled spaces between (111) layers of spheres.^47^

In the case of close-packed fcc SiO_2_ opals, the preceding scenario collapses (Figure 3d), and substitution of the fluid matrix by a solid produces an enhancement of the effective-medium linear slope, which is very close to the experimental one. The theoretical dispersion plot in Figure 3e predicts the LA branch in good agreement with the experiment, including bending close to the BZ edge. In this frequency region (at about 0.45 in reduced units), a bandgap region merging both the HG of dipole (*l* = 1) resonances and BG occurs. The experimental picture confirms that trend (see Figure 1d for several SiO_2_ opals with different diameters) where a very wide frequency region extending from 0.5 to 1.1 appears free of modes. For this system, many modes are theoretically expected above the LA bending frequency. Only very high mass-density contrast combinations (like metallic particles in polymer host) can provide huge hybridization gaps.^44^ We have also examined the inverse-topology scheme (Figure 3f), which badly fails, as it overestimates *c*_eff_ by a factor of 2 in spite of the adequate description of the HG frequency and width.

The reduced band diagrams of the low (Figure 3b) and high (Figure 3e) impedance contrast colloidal crystals reveal both similarities and distinct differences. In both cases, the assumption in the theoretical calculations of a solid PDMS host supporting shear waves in the interstitial space between spherical particles seems to successfully describe the first acoustic branch observed in our BLS experiments. Subsequently, a dipole-origin (*l* = 1) HG is predicted in both systems at frequencies just above this branch. In the case of the high elastic impedance SiO_2_ opal, the predicted HG coincides with a BG (Figure 3e). For the low-impedance soft colloids, the experimentally observed BG (Figure 2) is, however, not theoretically predicted under this solid host assumption (Figure 3b). An inverse-topology scheme (Figure 3c) succeeds in capturing the experimental BG qualitatively. These results suggest that solidification effects constitute an essential parameter in these systems that should not be neglected. On the other hand, the elastic impedance mismatch of the constituents strongly impacts the nature, frequency position, and width of the bandgaps. An attempt to consider intermediate values for the elastic mismatch between the scatterers and the host medium surrounding them is offered by hybrid particles combining hard cores and softer shells. We shall examine SiO_2_–PMMA core–shell colloidal crystals with the same core but different shell thicknesses and hence SiO_2_ volume fraction ϕ_SiO2_ of the nanoparticle (Table 1) in the next section.

### Core–Shell Colloidal Crystals

**Figure 4:**
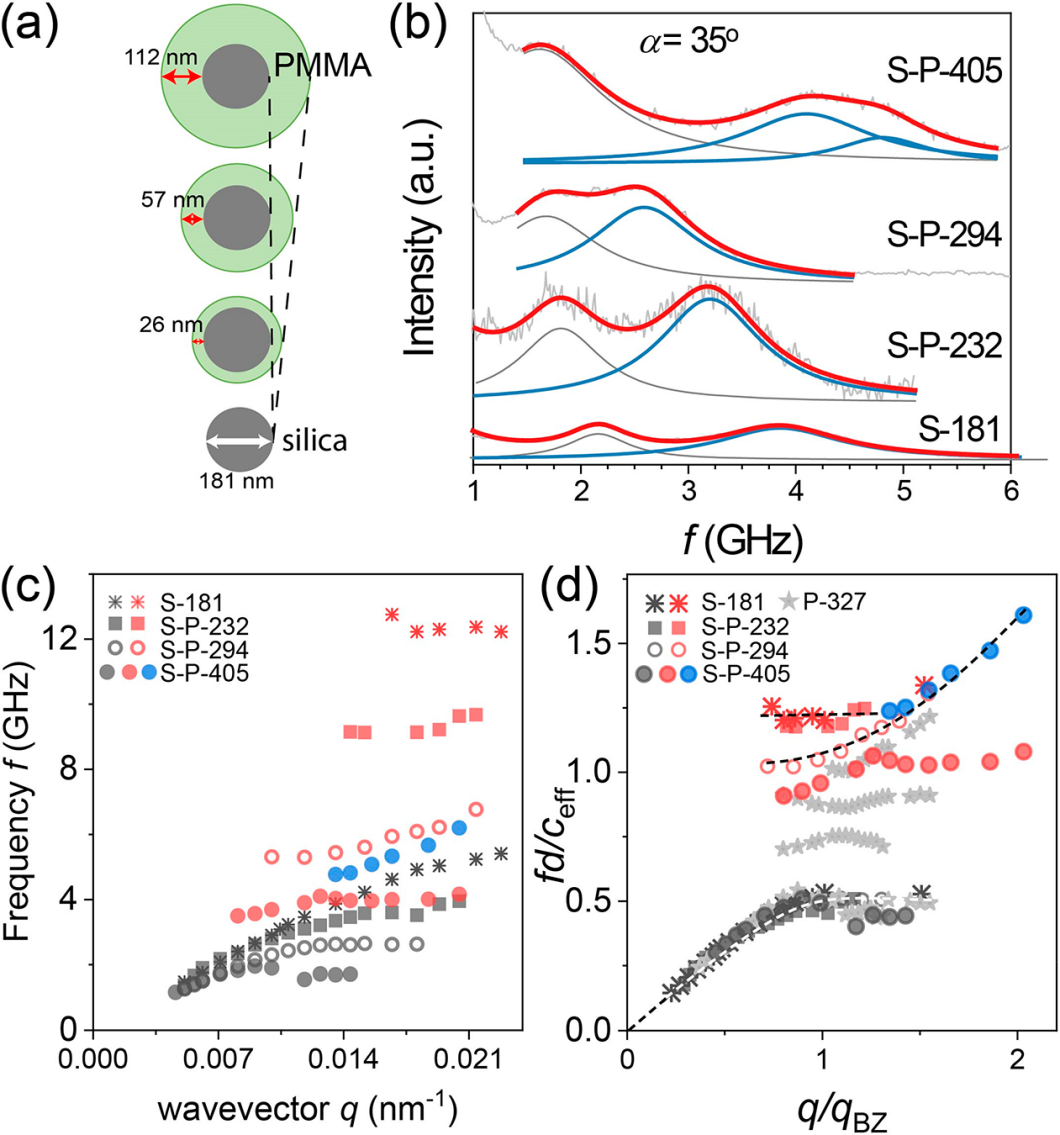
Phononic band diagrams of core–shell SiO_2_–PMMA crystalline films. (a) Schematic of core–shell SiO_2_–PMMA particles with increasing PMMA shell from 26 to 112 nm for the same silica core (*d*_c_ = 181 nm). (b) Exemplary anti-Stokes BLS spectra of SiO_2_–PMMA opals infiltrated with PDMS at the same incident angle, α = 35°. The spectra (gray lines) are represented by up to three Lorentzian curves (blue and gray lines) of which the lowest-frequency ones (gray lines) are PDMS modes. (c) Dispersion relations *f*(*q*) of wet opals of bare silica and SiO_2_–PMMA in a PDMS matrix. The corresponding normalized dispersion relations, *fd*/*c*_eff_ vs *q*/*q*_BZ_, along with the wet opal of PMMA (*d* = 327 nm) in the PDMS matrix^19^ are shown in panel d, where *d*, *c*_eff_, and *q*_BZ_ denote the particle diameter, effective-medium sound velocity, and the edge of first Brillouin zone (BZ) in the ΓM direction. The dashed lines are guides to the eye.

Figure 4a shows schematically three SiO_2_–PMMA core–shell nanoparticles with ϕ_SiO2_ varying between 1 and 0.09 (Table 1). For comparison, BLS spectra for the three core–shell colloidal crystals and the parent S-181, all infiltrated with PDMS, are shown at *q* = 0.0135 nm^–1^ in Figure 4b. The different LA phonon frequency at constant *q* is also seen in the dispersion plot of Figure 4c and is due to the dependence of the linear acoustic regime on the lattice constant and ϕ_SiO2_. In fact, this LA branch superimposes for all S-P, S, and PMMA systems in the reduced dispersion diagram of Figure 4d. However, less successful is the superposition of the high-frequency branch, which seemingly depends on ϕ_SiO2_. For S-181 (ϕ_SiO2_ = 1) and S-P-232 (ϕ_SiO2_ = 0.47), the upper branch is insensitive to composition (flat dashed line in Figure 4d), and the band diagram is SiO_2_-like up to 53% PMMA shell fraction. Upon further increase of the PMMA shell (and hence *d*), the bandgap narrows as seen by the red frequency shift of the upper branch in Figure 4d, and *c*_eff_ seemingly drops from 1800 to 1530 m/s (Table 1). However, the concurrent decrease of *f*_bend_ for the LA mode reduces the *q*-range of linear acoustic behavior rendering an underestimation of *c*_eff_ with an increasing core–shell particle diameter. Nevertheless, the successful superposition of the LA branch for the four opals in Figure 4d suggests that *f*_bend_ follows *c*_eff_ and the particle diameter. In fact, *f*_bend_*d*/*c*_eff_ ∼ 0.5 for the three core–shell and S-181 opals (see Figure 3e) in agreement with Figure 4d. The band diagram remains distinct from that of PMMA colloidal crystal (gray stars) even up to 91% PMMA shell fraction (S-P-405) (solid circles) with no indication of BG as observed in PMMA opals (Figure 2). This nonmonotonic dependence of the band diagram on the soft PMMA shell fraction, the lack of superposition of the upper branch, and the absence of BG are novel and unexpected findings, which are theoretically addressed next.

**Figure 5:**
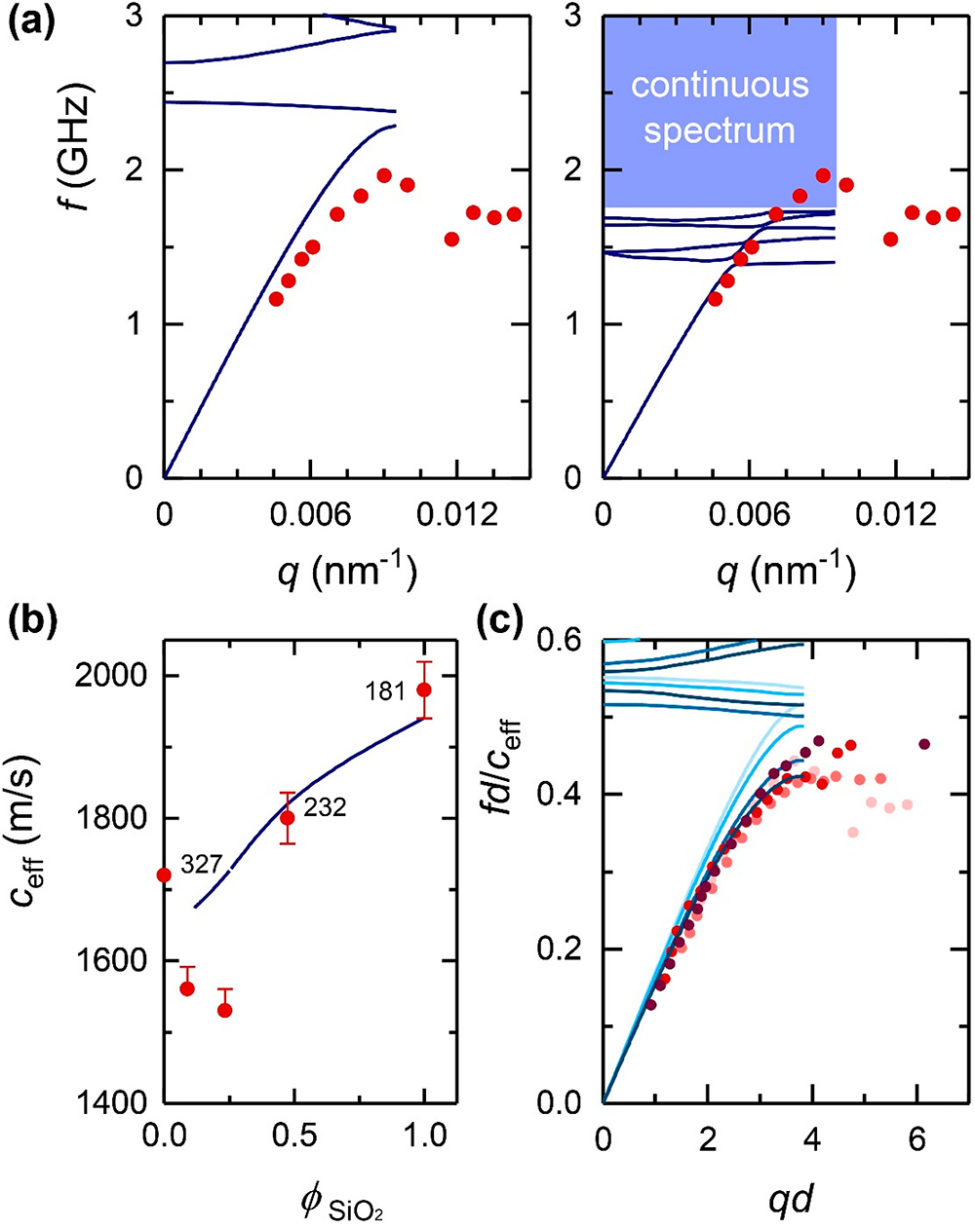
(a) Theoretical dispersion for longitudinal bands (solid lines) for fcc arrays of close-packed core–shell SiO_2_–PMMA particles (S-P-405) with diameter *d* = 405 nm (silica core diameter *d*_c_ = 181 nm) infiltrated with PDMS considered solid with *c*_T_ = 400 m/s (left panel) and *c*_T_ = 200 m/s (right panel). In both cases, *c*_L,PDMS_ = 1050 m/s. Symbols denote the experimental data. (b) Variation of the experimentally deduced *c*_eff_ with the silica volume filling fraction, ϕ_SiO2_, for the core–shell samples and the bare core silica opal, S-181. The solid line denotes the simulated *c*_eff_ for the four systems. (c) Reduced band diagram for all hybrid samples, including bare silica opals, with darker lines and symbols corresponding to thinner PMMA shells.

Guided by the rather successful description of the component systems (Figure 3) in an effectively solid PDMS host (*c*_T,PDMS_ = 400m/s), we first assumed the same methodology. Starting with the bare silica crystal (S-181 opal), the progressive increase of the PMMA shell has revealed a still coherent description only in the case of the S-P-232 crystal (see Figure S3). For the samples S-P-294 and S-P-405, the solid host assumption in the theoretical calculations fails to capture both *c*_eff_ and the bending of the LA branch. Both quantities clearly overestimate their experimental counterparts suggesting weaker interparticle interactions with increasing size that can be effectively described by lowering the *c*_T,PDMS_ value. Figure 5a presents the theoretical band diagram for the core–shell colloidal crystal with diameter *d* = 405 nm (S-P-405) using *c*_T,PDMS_ = 400 m/s (left panel) and *c*_T,PDMS_ = 200 m/s (right panel); the same plot for S-P-294 using *c*_T,PDMS_ = 400 and 250 m/s is shown in Figure S4. As seen in Figure 5a and Figure S4, the two core–shell systems with ϕ_SiO2_ < 0.5 show a more liquid-like matrix behavior manifested as a decrease of both *c*_eff_ and *f*_bend_. It is worth noting that, for these two more dilute in silica samples, a clear drop of *c*_eff_ is observed in Figure 5b, which also implies an underestimation of the experimental values obtained from Figure 4c. Note that the theoretical values of *c*_eff_ refer strictly to the long-wavelength limit, which is however not accessible in the experimental dispersion (Figure 4c), in particular for the two systems with large (294 and 405 nm) diameters. Although the drop of *c*_eff_ for these two samples could be captured by the assumption of a liquid-PDMS host, the latter still fails to represent the bending frequency of the LA (see Figure S5) as for the component colloidal crystals in Figure 3a,d.

In view of the successful superposition of the experimental LA diagrams in Figure 4d, we examine the same reduced diagram for the theoretical curves in Figure 5a. The overlap in the long-wavelength acoustic regime is trivial, but the bending region is less successfully superimposed, as seen in Figure 5c. Given the higher resolution of the latter compared to Figure 4d, the solid–solid boundary conditions are still a better approximation for all four samples compared to the solid–liquid case in Figure S5. The above behavior can be possibly understood as a scale-dependent effect assuming homogeneous interparticle contacts. An increase of the PMMA-shell thickness with a constant-diameter core (S-181) increases the lattice constant and hence the size of the interstitial cavities that cannot be represented by an effective PDMS solidification. In the same context, adhesive contacts between these polymer-shell particles in the liquid-PDMS matrix may become less dominant, leading to the drop of *c*_eff_ (Figure 5b), suggesting a transition at some scale. Notably, the value *c*_eff_ (=1720 m/s) in the PMMA (*d* = 327 nm) opal in PDMS (Figure 2) is higher than in S-P-294 and S-P-405 and follows the trend for S-P-232 and S-181 in Figure 5b. The observed overestimation of the *c*_eff_ with decreasing ϕ_SiO2_ might hint to a different PMMA packing in the shell and/or the effective PDMS elasticity due to the increase of the size of the interstitial cavities. We recall that the phonon dispersion in these three crystals was represented using the same *c*_T,PDMS_ value (=400 m/s). The PMMA chain conformation and surface mobility,^42^ which impact interparticle interactions, are different in the bold and core–shell particles infiltrated with PDMS. Change of the PMMA packing can impact *c*_eff_ as recently reported in the case of polymer grafted SiO_2_.^48^ However, this delicate conformation issue merits a detailed study in the future.

According to the theoretical calculations, the nature of the bandgap in SiO_2_ and SiO_2_–PMMA is a merged BG and HG (Figure 5a). From the experimental side, we examine the nature of the bandgap in SiO_2_–PMMA opals introducing disorder by mixing S-P-294 and S-P-405 with three different volume ratios. The three hybrid colloidal glasses are disordered according to the SEM images as shown for example in Figure S6 for 1:3 A/B where A and B denote the small S-P-294 and large S-P-405, respectively. The experimental dispersion diagrams of the two hybrids, 1:1 and 1:3, infiltrated with PDMS, as shown in the right panel of Figure S7, along with those for the A and B colloidal crystals, display a single bandgap which is robust to disorder. This finding corroborates the notion of an HG as also implied by the comparison to the PMMA colloidal crystal in Figure 4d. Since the elasticities of the SiO_2_ in A and B are the same within error (Table 1), the moderate superposition of the acoustic branch in the left panel of Figure S7 is due to the different spacing in the three disordered hybrids. An attempt to superimpose this branch with that in S-P-405 would require spacing 370 and 405 nm for the 1:1 and 1:3 hybrid, respectively (Table S1).

## Conclusions

We have utilized SiO_2_ (hard), PMMA (soft), and SiO_2_–PMMA core–shell colloidal crystals infiltrated with PDMS to record the hypersonic phonon propagation far from and near to the Brillouin zone by BLS. New unexpected experimental findings and possible explanations emerge from the comparison with the theoretical calculation of the band diagrams of the systems with variable elastic impedance contrast.

The effective-medium sound velocity *c*_eff_ obtained from the long-wavelength region of the dispersion relation is larger than the predicted value for hard phononic crystals in a liquid (PDMS) matrix. Moreover, *c*_eff_ drops significantly in a SiO_2_–PMMA colloidal crystal below 50% SiO_2_ filling fraction in the particle. Both findings are rationalized by assuming interparticle contacts and therefore solid–solid-like phononic behavior. The latter is parametrized by an effective transverse sound velocity of the matrix PDMS that varies between 200 and 400 m/s with an increased SiO_2_ fraction.

The solid–solid phononic nature impacts the origin of the particle vibration resonance-induced hybridization stopband, which changes from the quadrupolar (*l* = 2) to dipole (*l* = 1) particle resonance in both SiO_2_ (hard) and PMMA (soft) colloidal crystals. The latter exhibits an additional high-frequency order-induced BG, whereas there is no indication of a BG in the theoretical band diagram along high-symmetry directions. In the case of SiO_2_ and SiO_2_–PMMA opals, there is a single bandgap, which is a mixed HG and BG in the theoretical dispersion of the SiO_2_ colloidal crystal. The HG nature of this bandgap was verified in the case of disordered SiO_2_–PMMA systems.

Scalability of the band diagrams, indicative of system-dependent behavior, is verified for the bare SiO_2_ and PMMA, while for the SiO_2_–PMMA colloidal crystals it works only for the low-frequency longitudinal acoustic branch. The upper branch is composition-dependent, as interfacial connectivity and variation of the particle elasticity with the core composition are not captured in the reduced presentation, *fd*/*c*_eff_. This study shows that the theoretical prediction of the experimental band diagram of the colloid-based hypersonic colloidal crystals is still incomplete and merits further detailed exploitation in the future. On the experimental side, a systematic variation of the colloid eigenfrequencies, its filling ratio, and components elastic mismatch would help scrutinize the origin of the HG characteristics for acoustic metamaterials.

## Supporting Information Available

Theoretical density-of-states calculations of the quadrupole spheroidal mode of a spherical silica particle, inverse-topology scheme for the description of almost touching spherical particles in cross-linked PDMS, theoretical dispersion for longitudinal bands for fcc arrays of close-packed core–shell particles, and SEM image and dispersion relations of disordered hybrid colloidal crystals (PDF)
