# Supplementary material for: Origin of the Acoustic Bandgaps in Hypersonic Colloidal Phononics: The Role of the Elastic Impedance
Source: J Phys Chem B. 2022 Aug 23;126(34):6575–84. doi: 10.1021/acs.jpcb.2c03923 (PMC9442645; doi:10.1021/acs.jpcb.2c03923)
Supplement: Supplementary file 1 — jp2c03923_si_001.pdf [file jp2c03923_si_001.pdf]

Supporting Information

Origin of the Acoustic Bandgaps in Hypersonic Colloidal Phononics: The Role of the Elastic Impedance

Yu Cang[1,2], *Rebecca Sainidou*[3], *Pascal Rembert*[3], *Giulia Magnabosco*[4], *Tim Still*[1], *Nicolas Vogel*[4], *Bartlomiej Graczykowski*[1,5], *George Fytas*[*1,6]

[1]Max Planck Institute for Polymer Research, Ackermannweg 10, 55128 Mainz, Germany

[2]School of Aerospace Engineering and Applied Mechanics, Tongji University, Zhangwu Road 100, Shanghai 200092, China

[3]Laboratoire Ondes et Milieux Complexes UMR CNRS 6294, UNIHAVRE, Normandie University, 75 rue Bellot, F-76600 Le Havre, France

[4]Institute of Particle Technology, Friedrich-Alexander University Erlangen-Nürnberg, 91058 Erlangen, Germany

[5]Faculty of Physics, Adam Mickiewicz University, Uniwersytetu Poznanskiego 2, Poznan 61-614, Poland

[6]Institute of Electronic Structure and Laser, FO.R.T.H, N. Plastira 100, /0013, Heraklion, Greece

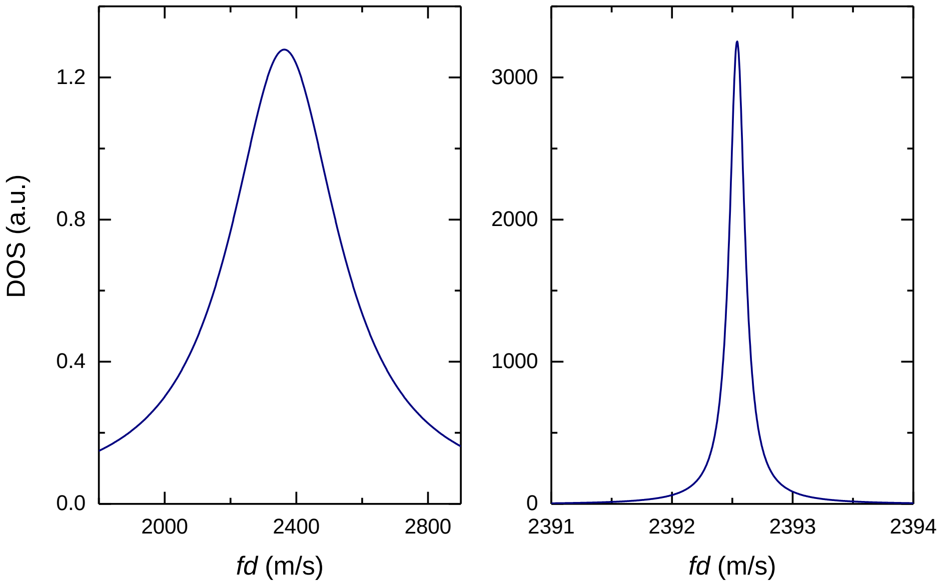

Figure S1. Theoretical density-of-states (DOS) calculations of the quadrupole (1,2) spheroidal mode of a spherical silica particle ($c_{L,SiO2}$= 4540 m/s, $c_{T,SiO2}$= 2860 m/s and ρ=1900 kg/m^3) of diameter d embedded in PDMS host, assumed fluid (left plot), and in air (right plot). The resonance peak occurs at $\boldsymbol{fd = 2362}$ m/s for the PDMS host, and is slightly blue-shifted ($\boldsymbol{fd = 2392.5}$ m/s) when air surrounds the sphere.

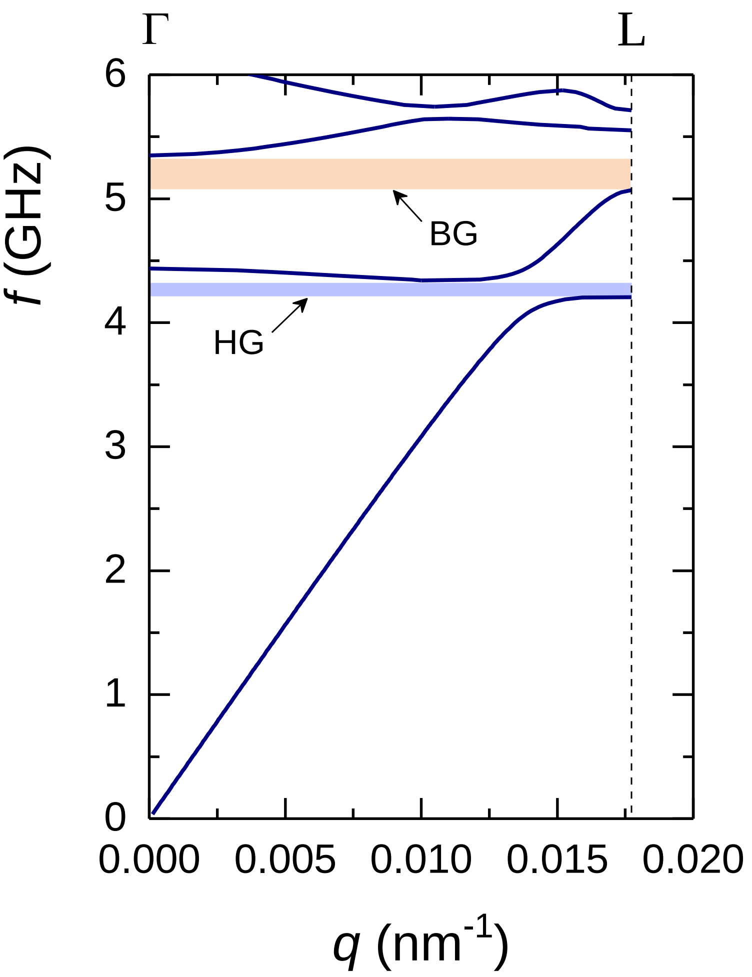

Figure S2. Inverse topology scheme for the description of almost touching PS spherical particles in crosslinked PDMS, arranged in an fcc lattice, studied in Ref [1]. The theoretical band diagram (only longitudinal bands are shown) assumes fcc array (lattice constant $a = 307\,nm$) of solid PDMS ($\rho = 965\,kg\,m^{-3}$, $c_L = 1190\,m\,s^{-1}$, $c_T = 500\,m\,s^{-1}$) spherical inclusions (diameter $d = 157nm$) in a PS matrix ($\rho = 1050\,kg\,m^{-3}, c_L = 2350\,m\,s^{-1}$, $c_T = 1200\,m\,s^{-1}$), along ΓL direction. The hatched stripes indicate the periodicity induced Bragg gap (BG) and the hybridization bandgap (HG) associated to dipole PDMS resonances.

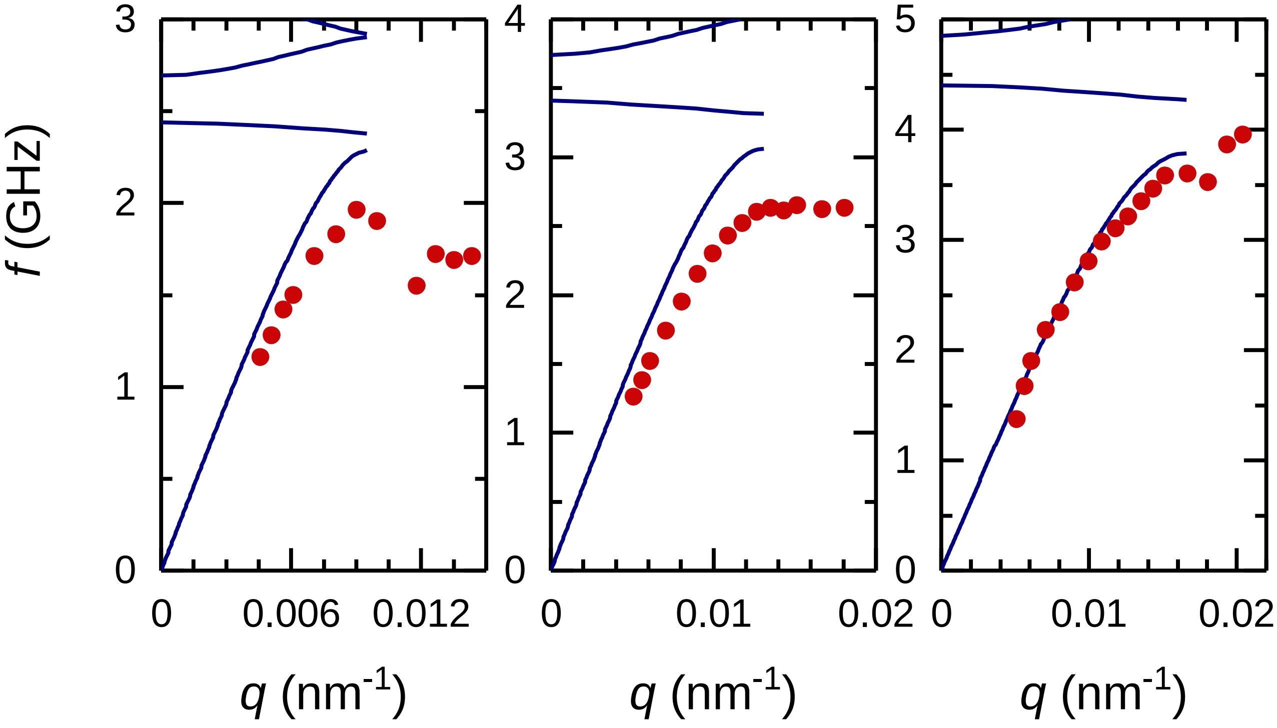

Figure S3. Theoretical dispersion for longitudinal bands (solid lines) for fcc arrays of close-packed core-shell SiO2-PMMA particles (silica core diameter d_c=181 nm) infiltrated with PDMS considered solid (c_L=1050 m/s, c_T=400 m/s) for three different PMMA shell thicknesses (particle diameter d=405nm, 294nm and 232nm from left to right). Symbols denote the experimental data.

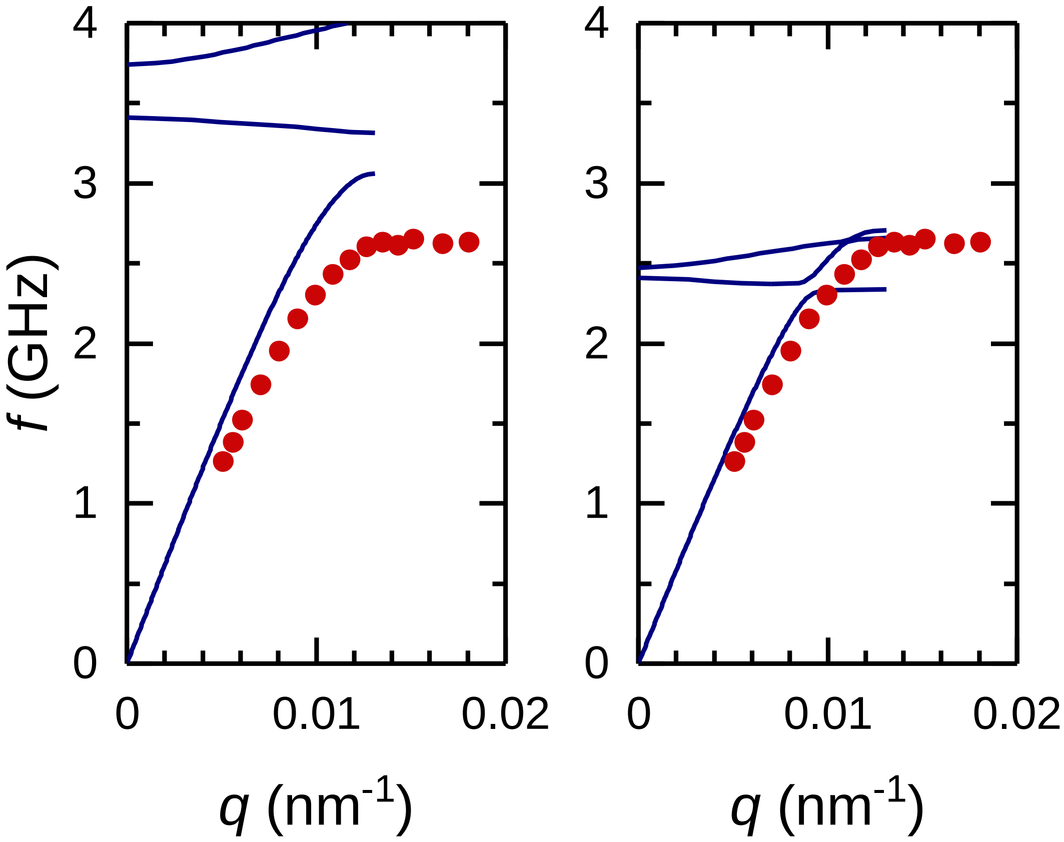

Figure S4. Theoretical dispersion for longitudinal bands (solid lines) for fcc arrays of close-packed core-shell SiO2-PMMA particles (silica core diameter d_c=181 nm) with diameter 294 nm infiltrated with PDMS considered solid using either c_T=400 m/s (left) and c_T=250 m/s (right). Symbols denote the experimental data.

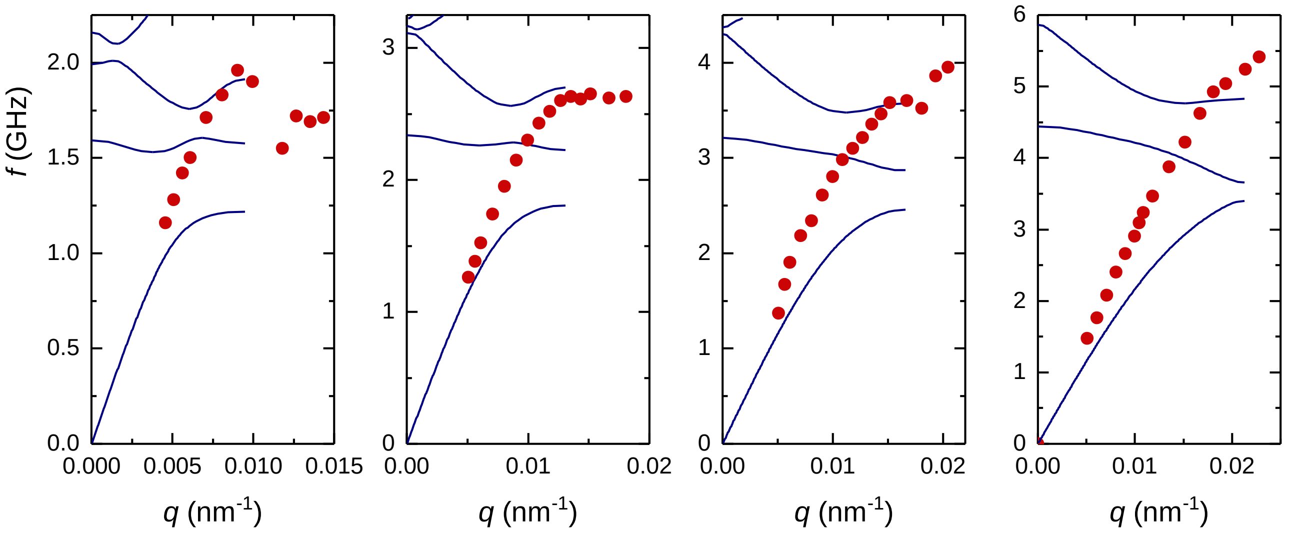

Figure S5. Phonon dispersion in three SiO_2-PMMA core-shell (particle diameter d = 405nm, 294nm and 232nm from left to right) and SiO_2 (S-181) colloidal crystals infiltrated in PDMS. In contrast to Fig.5a the theoretical dispersion diagrams (solid lines) were computed assuming liquid PDMS that captures the experimental c_{eff} only for S-P-405 and S-P-294 opals with high PMMA content. Symbols denote the experimental data.

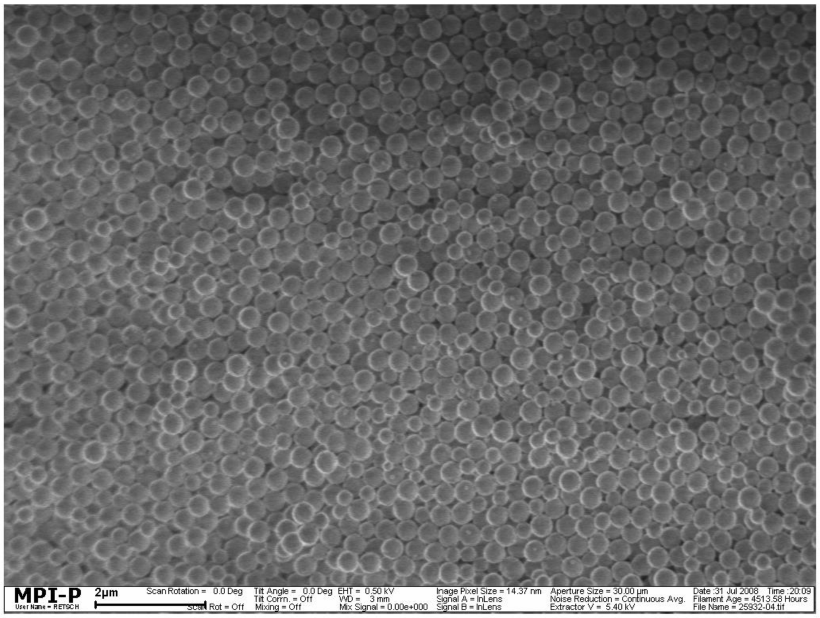

Figure S6. SEM image of the disordered hybrid colloidal crystal with S-P-295 and S-P-405 at a volume ratio of 1:3.

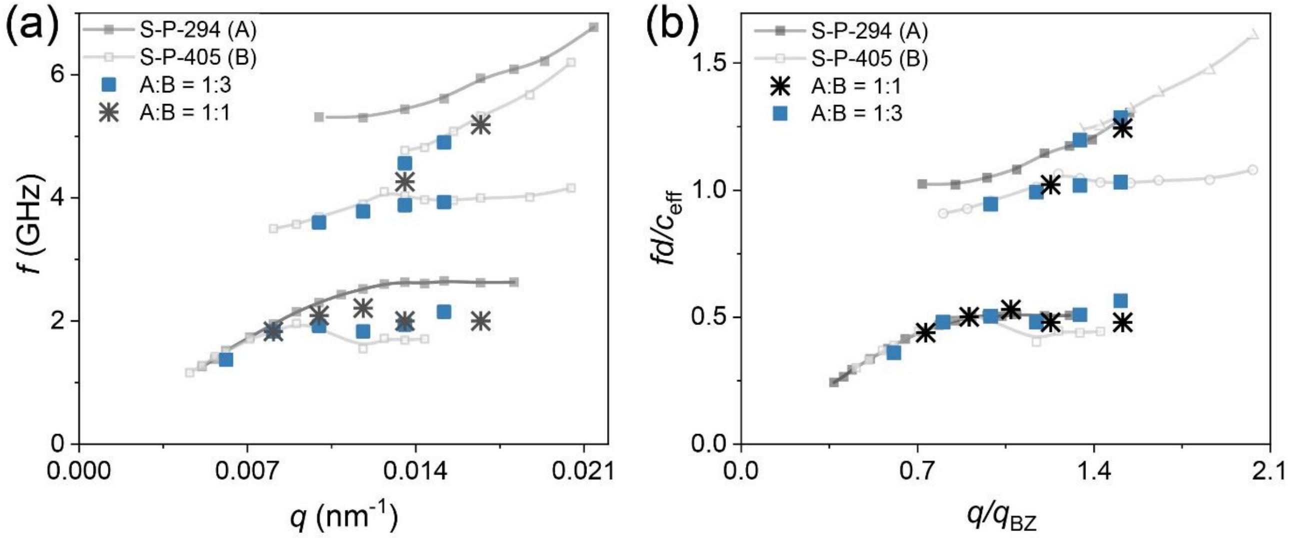

Figure S7. Phononic band diagrams of disordered wet films with hybrid particles of S-P-294 and S-P-405. (a) Dispersion relations of wet opals of hybrid particles in the PDMS matrix along with that of the individual S-P-294 and S-P-405 particles. The corresponding normalized dispersion relations, fd/c_{eff} vs. q/q_{BZ}, are shown in (b), where the d and c_{eff} used for two hybrids are listed in Table S1 and $q_{BZ} = 3\sqrt{3}\pi/4d$ is the edge of the first Brillouin zone (BZ) in the ΓM direction.

Table S1. Spacing *a*, and effective sound velocity, c_{eff} of two hybrids.

Volume ratio of Hybrids S-P-294 (A): S-P-405 (B)	Average particle diameter *d** (nm)	c_{eff}# (m/s)
1:1	370	1543
1:3	405	1543

*Average particle diameter *d* is used for hybrid to superimpose its normalized dispersion relation to S-P-405.

#c_{eff} is estimated from the linear fit at low ***q*** regime.

References

1. Zhu, G. H.; Swinteck, N. Z.; Wu, S. T.; Zhang, J. S.; Pan, H. H.; Bass, J. D.; Deymier, P. A.; Banerjee, D.; Yano, K., Direct Observation of the Phonon Dispersion of a Three-Dimensional Solid/Solid Hypersonic Colloidal Crystal. *Physical Review B* **2013,** *88* (14), 144307.
